# SAPHO syndrome with a purely osteolytic form: A case report

**DOI:** 10.1016/j.radcr.2023.07.034

**Published:** 2023-08-03

**Authors:** Akira Kakiuchi, Yayoi Yamamoto, Akira Kanbe, Daichi Kojima, Ayumi Horikawa, Ayako Hino, Tsunehiro Doiuchi, Hiroaki Kurihara

**Affiliations:** Department of Diagnostic and Interventional Radiology, Kanagawa Cancer Center, 2-3-2 Nakao Asahi-ku, Yokohama, Kanagawa, Japan

**Keywords:** SAPHO syndrome, Osteitis

## Abstract

SAPHO (synovitis, acne, pustulosis, hyperostosis, and osteitis) syndrome is a rare, chronic autoinflammatory disorder that can present with a constellation of cutaneous and osteoarticular symptoms. Osteodestructive lesions are not pathognomonic, whereas hyperostosis and osteitis are the most prominent imaging findings. We report the case of a man with osteolytic changes of the lumbar vertebra and a history of palmoplantar pustulosis. Biopsy revealed no neoplasm, suggesting SAPHO syndrome. Our case demonstrates that knowledge of atypical radiologic findings is necessary for the diagnosis of SAPHO syndrome.

## Introduction

SAPHO (synovitis, acne, pustulosis, hyperostosis, and osteitis) syndrome is a rare chronic autoinflammatory disorder of unknown etiology characterized by a combination of cutaneous and osteoarticular manifestations.

Osteoarticular radiologic findings of sclerotic changes or mixed sclerotic and osteolytic changes of bone in the anterior chest wall and spine are typical of this disease, but sometimes it is difficult to distinguish it from neoplasm [1].

Here, we present a case of SAPHO syndrome with osteolytic changes that were suspicious for neoplasm.

## Case

**Fig. 1:**
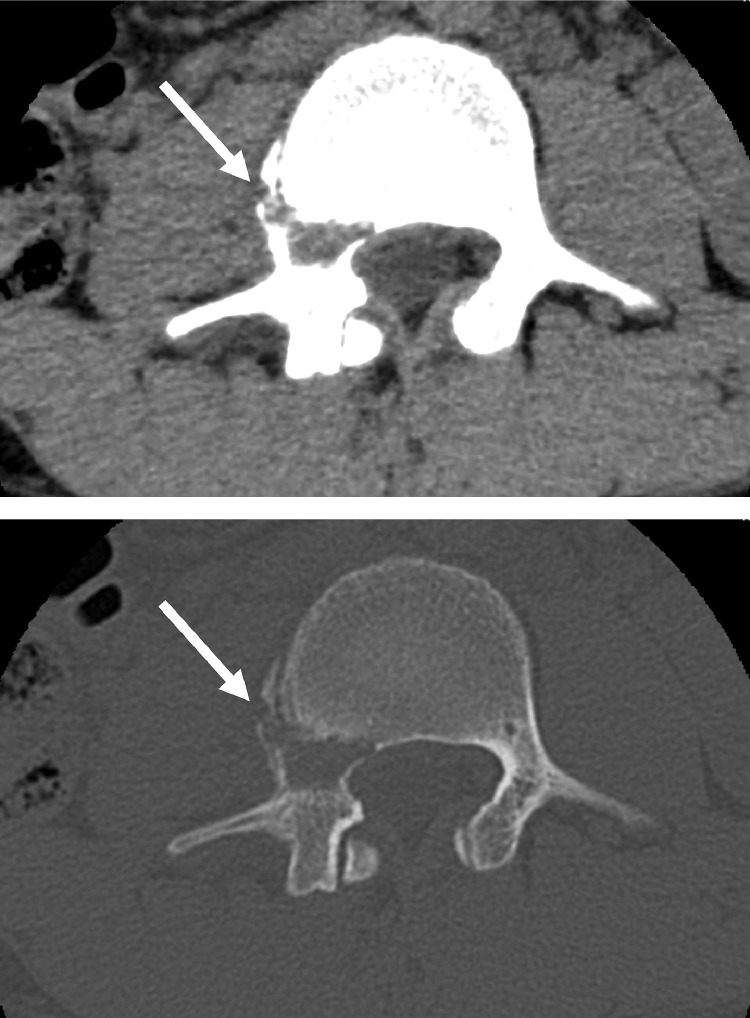
Computed tomography scan showing osteolytic changes in the right pedicle of the third lumbar vertebra.

**Fig. 2:**
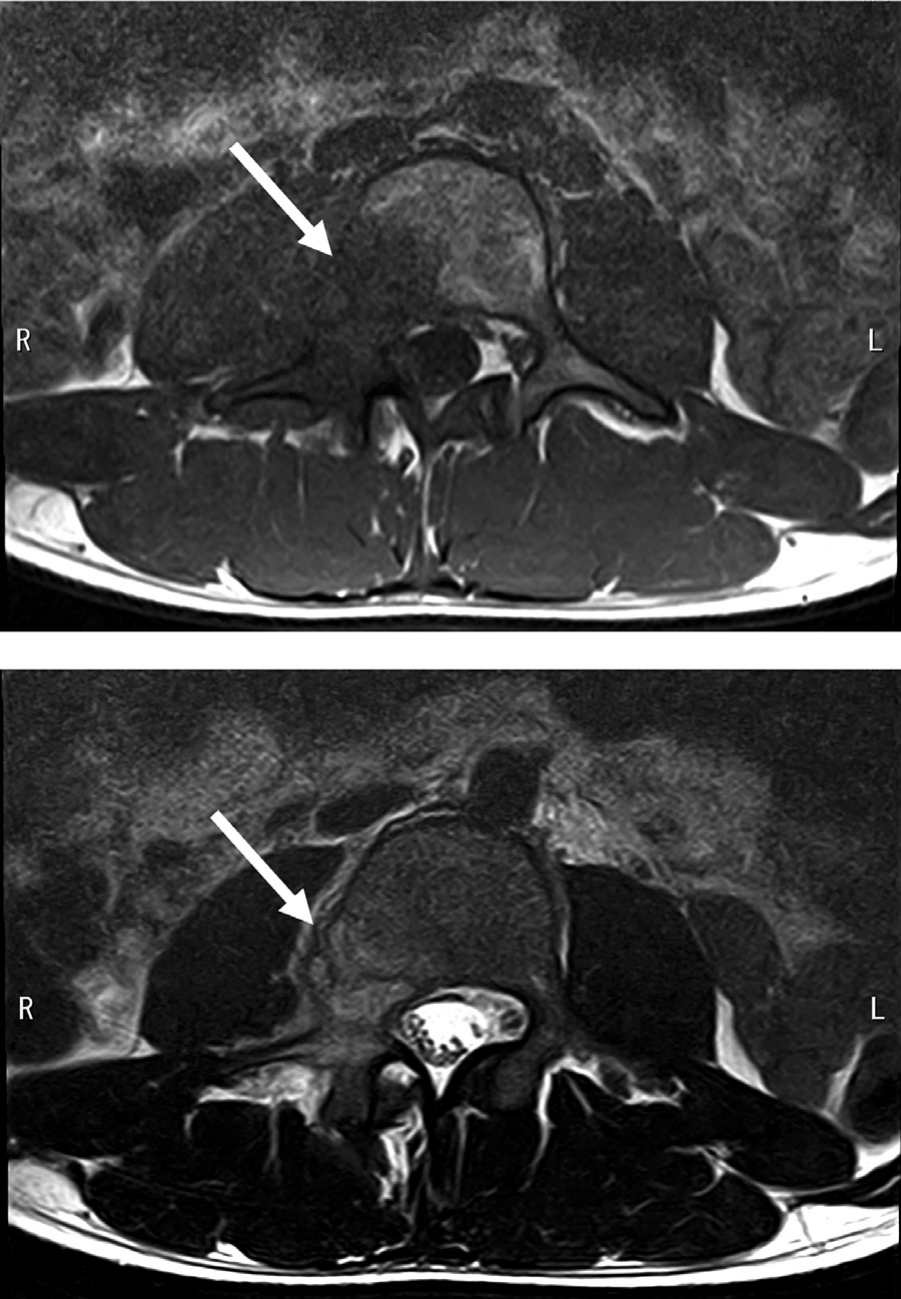
Axial T1WI imaging showing low signal intensity and T2WI imaging showing high signal intensity, suggestive of a neoplastic lesion.

**Fig. 3:**
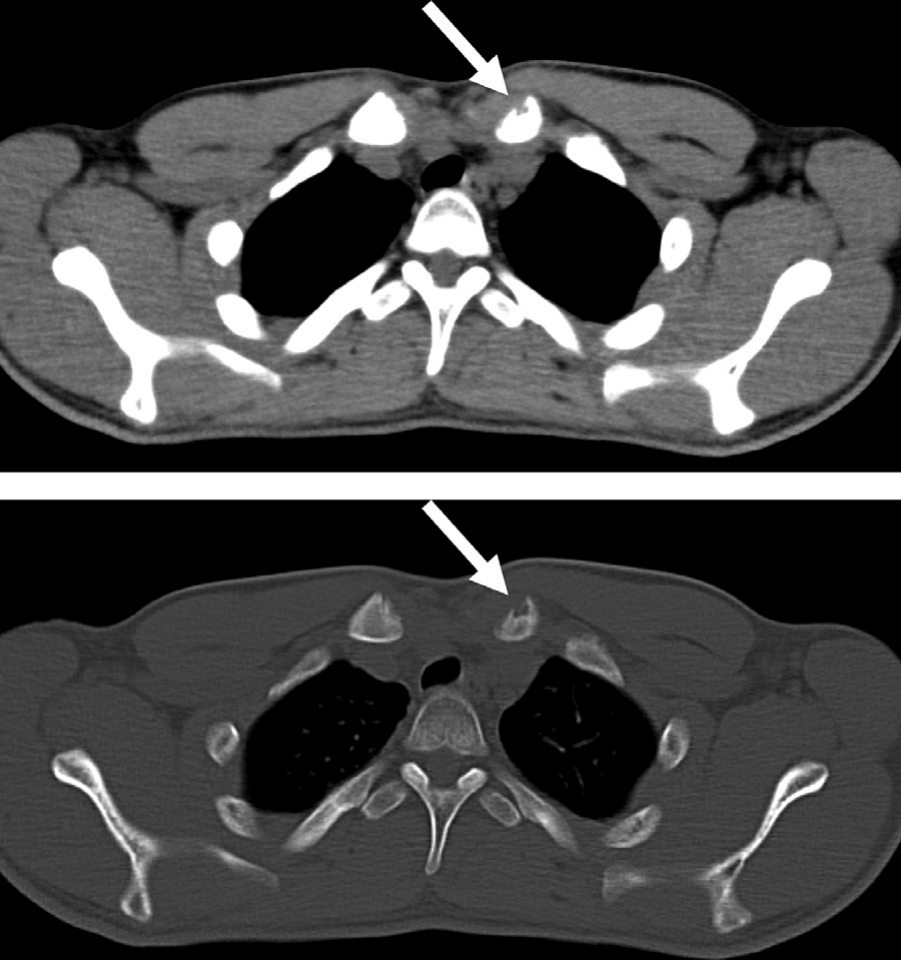
Computed tomography scan showing small osteolytic changes in the left clavicle, consistent with SAPHO syndrome.

A 19-year-old man presented with signs and symptoms suspicious of lumbar spine neoplasm. He had a 3-week history of worsening back and right leg pain. Four weeks before admission, he received dental treatment and was prescribed nonsteroidal anti-inflammatory drugs (NSAIDs). After taking the NSAIDs, a skin rash developed. The skin lesion presented as nodulocystic acne involving the distal upper and lower extremities, and was preliminarily diagnosed as drug-induced rash or palmoplantar pustulosis. Bacterial culture of the skin was negative. Blood tests showed a mildly elevated C-reactive protein level of 1.73 mg/dL. Computed tomography (CT) revealed osteolytic changes in the right pedicle of the third lumbar vertebra, but the sclerotic changes were unclear (Fig. 1). Magnetic resonance imaging (MRI) showed low intensity on T1-weighted images and high intensity on T2-weighted images, and the areas of abnormal intensity were more extensive than those of the changes seen on CT, including the right facet joint and vertebral body (Fig. 2). Although SAPHO syndrome may be suspected based on palmoplantar pustulosis, the lumbar spine lesion strongly suggested a neoplasm such as giant cell tumor because of its purely osteolytic features on CT. An open biopsy was performed under general anesthesia. The lesion was hard, and the osteolytic tumor was ruled out. Histopathological findings showed hyperplastic bone marrow with a prominent inflammatory cell infiltration, including neutrophils, eosinophils, and plasma cells. Neoplastic cells were not evident. Whole-body CT revealed small osteolytic changes in the left clavicle (Fig. 3). The final diagnosis was osteomyelitis. A clinical diagnosis of SAPHO syndrome was made based on both the skin and lumbar spine lesions. The patient was referred to a general hospital for further management.

## Discussion

The patient, in this case, was diagnosed with SAPHO syndrome because even though the bone changes were purely osteolytic, the skin manifestations of palmoplantar pustulosis and hyperostosis met the diagnostic criteria, with negative results on bacterial culture.

SAPHO syndrome is a rare, chronic, autoinflammatory disorder of unknown etiology. There are at least 3 sets of diagnostic criteria for SAPHO syndrome, but none has been clinically validated [2] (Table 1). Osteoarticular lesions of SAPHO syndrome include synovitis, hyperostosis, osteitis, arthropathy, and enthesopathy, which may occur in the anterior chest wall (65%-90%), spine (33%), pelvis (13%-52%), and long bones (30%) [3]. Hyperostosis and osteitis are the most prominent imaging findings in SAPHO syndrome, and osteolytic lesions are not radiologically characteristic [3,4]. Purely osteolytic lesions are rare. Kinoshita et al. [5] reported that X-rays of the femur in a patient with SAPHO syndrome showed changes from a purely osteolytic lesion to an osteosclerotic lesion during a 15-month follow-up. Osteoarticular lesions tend to be osteodestructive in early stages and osteoproliferative in later stages [1]. Our case may represent the very early phase of osteoarticular SAPHO syndrome.

**Table 1:** Three existing sets of diagnostic criteria for SAPHO syndrome.

Benhamou et al. [6]	Kahn and Khan [7]	Kahn [8], [9]
At least 1 of the following 4 conditions: 1. Osteoarticular manifestations of acne conglobate, acne fulminans, or hidradenitis suppurativa 2. Osteoarticular manifestations of PPP 3. Hyperostosis (of the ACW, limbs or spine) with or without dermatosis 4. CRMO involving the axial or peripheral skeleton, with or without dermatosis	At least 1 of the following 3 conditions: 1. Chronic, recurrent multifocal, sterile axial osteomyelitis, with or without dermatosis 2. Acute, subacute, or chronic arthritis associated with PPP, pustular psoriasis, or severe acne 3. Any sterile osteitis associated with PPP, pustular psoriasis, or severe acne	At least 1 of the following 5 conditions: 1. Bone–joint involvement associated with PPP and psoriasis vulgaris 2. Bone–joint involvement associated with severe acne 3. Isolated sterile hyperostosis or osteitis 4. CRMO (children) 5. Bone–joint involvement associated with chronic bowel disease Exclusions: Infectious osteitis, tumoral conditions of bone, non-inflammatory condensing lesions of bone

ACW, anterior chest wall; CRMO, chronic, recurrent, multifocal osteomyelitis; PPP, palmoplantar pustulosis; SAPHO, synovitis, acne, pustulosis, hyperostosis, and osteitis.

In addition to the osteolytic changes in our case, the presence of a lesion on the pedicle was also atypical, because spinal lesions in SAPHO syndrome are generally characterized by corner lesions and nonspecific spondylodiscitis [1]. In making the diagnosis, it is important to rule out neoplastic bone disease or infectious osteitis. Histopathologic findings are necessary to exclude neoplastic disease. If characteristic skin lesions are present, SAPHO syndrome should be strongly considered in the differential, even if the lesions involve purely osteolytic changes. The presence of bone lesions in other areas, such as the anterior chest wall in this case, may increase confidence in the diagnosis of SAPHO syndrome and avoid the need for unnecessary biopsies.

In conclusion, we report a case of SAPHO syndrome with a purely osteolytic form. The early phase of osteoarticular SAPHO may be osteolytic. Awareness of the imaging appearances may help to consider a diagnosis of initial SAPHO syndrome in patients presenting with nonspecific bone conditions. Prompt diagnosis can avoid an unnecessary string of investigations and biopsies.

## Patient consent

Written informed consent was obtained from the patient for their anonymized information to be published in this article.
